# Cup and Disc Segmentation in Smartphone Handheld Ophthalmoscope Images with a Composite Backbone and Double Decoder Architecture

**DOI:** 10.3390/vision9020032

**Published:** 2025-04-11

**Authors:** Thiago Paiva Freire, Geraldo Braz Júnior, João Dallyson Sousa de Almeida, José Ribamar Durand Rodrigues Junior

**Affiliations:** UFMA/Computer Science Department, Universidade Federal do Maranhão, Campus do Bacanga, São Luís 65085-580, Brazil; durand@nca.ufma.br

**Keywords:** fundus image, segmentation, U-Net, composition backbone segmentation

## Abstract

Glaucoma is a visual disease that affects millions of people, and early diagnosis can prevent total blindness. One way to diagnose the disease is through fundus image examination, which analyzes the optic disc and cup structures. However, screening programs in primary care are costly and unfeasible. Neural network models have been used to segment optic nerve structures, assisting physicians in this task and reducing fatigue. This work presents a methodology to enhance morphological biomarkers of the optic disc and cup in images obtained by a smartphone coupled to an ophthalmoscope through a deep neural network, which combines two backbones and a dual decoder approach to improve the segmentation of these structures, as well as a new way to combine the loss weights in the training process. The models obtained were numerically evaluated through Dice and IoU measures. The dice values obtained in the experiments reached a Dice of 95.92% and 85.30% for the optical disc and cup and an IoU of 92.22% and 75.68% for the optical disc and cup, respectively, in the BrG dataset. These findings indicate promising architectures in the fundus image segmentation task.

## 1. Introduction

Glaucoma is a disease that includes a group of optic neuropathies characterized by the progressive loss of retinal ganglion cells and their axons. Its cause is directly linked to sensitivity to intraocular pressure. This variation in intraocular pressure can be linked to several factors, including age, race, family history, genetics, environmental factors, thin central cornea, and myopia. Thus, control of intraocular pressure is the only clinically controllable risk factor [1]. The most common chronic forms of glaucoma are usually asymptomatic until the more advanced stages of the disease. Individuals may experience advanced visual field loss and progress to complete vision loss [2].

Glaucoma is the most common cause of blindness in the world; it is estimated that 50% of glaucoma sufferers living in developed countries are unaware of their clinical condition, and these numbers may be higher in underdeveloped countries [3]. Estimates indicate that 60.5 million people were affected by primary open-angle glaucoma and primary closed-angle glaucoma in 2010 worldwide [4]. According to these estimates, in 2020, over 76 million people will be affected by this disease, with projections of more than 111 million cases in 2040 [2]. With these numbers, there are projections of spending USD 14.6 billion on medical treatments alone [1].

Furthermore, due to the high cost of implementation and maintenance, studies prove that, at least in developed countries, population screening programs using traditional methods of diagnosing glaucoma are unfeasible [3].

Fundus imaging, or retinography, is essential for diagnosing and monitoring retinal diseases in ophthalmology. These images are 2D captures of the posterior segment of the eye, including blood vessels, optic disc (OD), macula, and fovea, which aid in detecting features related to glaucoma. Biomarkers, biological markers, are quantifiable indicators of biological states used in medicine to monitor health, evaluate treatments, diagnose disease, and predict progression. Retinography is the standard examination for the initial detection of glaucoma. The specialist analyzes two essential structures: the optic disc (OD) and the optic cup (OC). The intention is to identify essential biomarkers, such as the cup-to-disc ratio (CDR) and the neuroretinal rim (ISNT quadrants) [5,6,7,8].

Because it has great potential for patient screening, telemedicine, and clinical examinations, health professionals already know about the panoptic ophthalmoscope. This is justified by its portability, easy data transfer, and compatibility with smartphones and data acquisition applications, making these devices easy image capture tools. However, compared to conventional ophthalmic equipment, images obtained with this equipment have lower resolution [9].

Deep learning methods have shown superior performance to other image segmentation strategies due to their ability to extract features automatically [10]. Given the need for high accuracy in ophthalmological diagnosis, where minor errors can lead to blindness, and considering the workload and human limitations, computer-aided diagnosis (CAD) based on deep learning has become crucial for detecting and examining eye diseases [11]. Thus, retinal image segmentation methods are essential for automatically analyzing these images, providing object masks for quantitative processing and pathological explanation [10].

Aiming to highlight the biomarkers obtained from the morphology of the optic nerve (disc and cup) and inspired by the backbone composition model, this work presents a deep neural network architecture that uses the backbone composition and a dual decoder structure to segment the optic nerve structures in images obtained by a smartphone coupled to an ophthalmoscope. The proposed method contributes to using backbone composition in the medical image segmentation task and a dual decoder structure for segmenting images with objects of interest belonging to two classes. The experiments demonstrated that the composition could generate stable results, even in fundus photographs of different natures, demonstrating its generalization capacity. Finally, with cost reduction through artificial intelligence, this architecture could track patients in regions where material and human resources are scarce, contributing to the early diagnosis of glaucoma in these environments.

## 2. Related Works

To delimit the boundaries of the OC and OD structures as precisely as possible, several works have approached this task in the most varied possible ways.

The U-Net architecture is the preferred choice in many works with a similar goal. Ref. [12] combines DenseNet with a fully convolutional neural network (FCN) in a U-shaped structure. A U-Net with Resnet-34 as an encoder was used in [13] as a two-stage architecture. Ref. [14] proposes a multi-resolution architecture combined with attention gate modules on U-Net. Ref. [15] inserts convolutional blocks with residual connections in the encoder and decoder and channel-wise attention blocks in the skip connections. Ref. [16] presents a modification of TransUnet called EE-TransUnet, inserting two blocks, the Cascaded Convolutional Fusion Block and the Channel Shuffling Multiple Expansion Fusion Block. Ref. [17] proposes a modification of U-Net, inserting residual and attention modules. Ref. [18] also modifies U-Net by inserting spatial and channel attention layers in the skip connections, combined with a dense dilated series convolution layer.

Other works propose architectures based on other neural network architectures. Ref. [19] uses adversarial learning in a three-stage architecture: ROI Extraction, Segmentation (based on Deeplabv3+ with MobileNet V2 encoder), and Patch-level Discriminator (to motivate the segmentation network to produce similar outputs for the source domain). Ref. [8] proposes an architecture based on Fast R-CNN, with the addition of a Boundary Attention Module (BAM). Ref. [20] presents a two-stage architecture with a Multi-scale encoder (C-Net) and a graph convolutional network as a decoder (G-Net). Ref. [21] proposes a methodology called Sector Association and Multi-Coordinate Transformation Fusion, which combines Cartesian and polar coordinate representations to generate segmentation masks. Ref. [22] uses a polar coordinate transformed input and HR-Net as the backbone. The modifications in HR-Net include two blocks in the decoder (Semantic Segmentation Branch and Deep Supervision with Gradient Boosting) and a contour reconstruction block.

Some approaches do not present a new architecture but suggest different optical disc and cup segmentation methodologies. Ref. [23] used an ensemble learning methodology that was implemented to evaluate five backbones: ResNet34, ResNet50, MobileNet, Inceptionv3, and DenseNet121. Ref. [24] applied Canny Filter as a post-processing step for detecting and dilating the edges of the segmented objects. Ref. [25] used domain adaptation, where the architecture is formed by two parallel networks based on U-Net, one teacher and one student. Ref. [26] addressed the problem as a single-source domain generalization problem, using contrastive learning.

A summary of related work is presented in Table 1. An analysis of the related works showed that only one study investigated the impact of ensemble learning on the segmentation of structures that highlight geometric biomarkers in fundus images, and even that study applied a more conservative ensemble learning model with multiple backbones and a majority voting system. Similar results are expected with a backbone combination model, with a minor effort to find the optimal model. No work addresses the segmentation of images obtained by a smartphone and an ophthalmoscope. This absence may be because few studies aim to segment OC and OD in this type of image.

## 3. Materials and Methods

**Figure 1:**
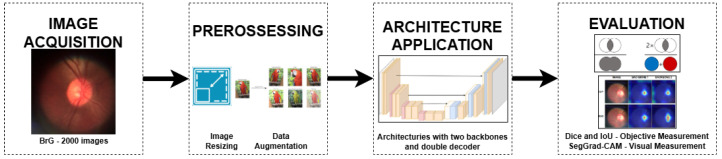
Methodology applied.

The methodology applied in this work is a deep learning image processing pipeline, with image acquisition, preprocessing, architecture construction, and evaluation results, as shown in Figure 1. The images from the BrG [9] dataset were used for image acquisition using the k-fold cross-validation methodology. The images were resized to 256 × 256 in preprocessing, and data augmentation techniques were applied from the training and validation groups. The deep learning architectures are designed with two backbones and a double decoder to obtain a refined delimitation of the region of interest. Finally, the results of the predictions on the images of the test folds were computed using the Dice and IoU measures.

### 3.1. Image Acquisition

The BrG [9] dataset is a set of exams obtained at the Hospital de Olhos, which has a glaucoma treatment program. The database consists of examinations of 1000 volunteers, 500 healthy individuals and 500 individuals with Glaucoma, with their right and left eyes photographed with a Welch Allyn 11820 Panoptic ophthalmoscope, generating a total of 2000 images. The panoptic ophthalmoscope was used because it is portable and easy to acquire images since it only requires a smartphone and exam acquisition software. Therefore, it is easy to share fundus images. The images in the dataset were obtained without using ophthalmic dilators. They have an approximate 25° field of view and are centered on the optic disc. Thus, the images available in the dataset are cutouts of the exams, approximately 400 × 400 pixels [9].

By analyzing the images in the dataset, it was observed that 372 images, with a normal subset, did not have the cup marking. Therefore, they were removed from the architecture evaluation process, resulting in only 1628 samples, 1000 images of glaucomatous eyes, and 628 of normal eyes.

### 3.2. Preprocessing

The fundus images were used with three channels. They were resized to a resolution of 256 × 256 pixels in the preprocessing process. This resizing was performed to meet the computational limitations while maintaining characteristics relevant to the delimitation of the structures in the images. All images were normalized before network input.

In addition to resizing, data augmentation was performed in the training set using the Albumentations [27], consisting of the following functions: HorizontalFlip, CLAHE (clip limit = 4.0, tile grid size = (8, 8)), AdvancedBlur (blur limit [3, 7]; sigma limit = [0.2, 1.0]; rotate limit = [−90, 90], beta limit = [0.5, 8.0], noise limit = [0.9, 1.1]), and RandomBrightnessContrast (brightness limit = [−0.7, 0.7], contrast limit = [−0.7, 0.7], brightness by max = True, ensure safe range = False), all with a 50% probability of applicability. These values were obtained empirically and were chosen based on geometrical alterations limited to horizontal flip (left eye and right eye), and others imagining that the image alterations at exams are brightness and contrast. And the images were not normalized.

### 3.3. Architecture Construction

**Figure 2:**
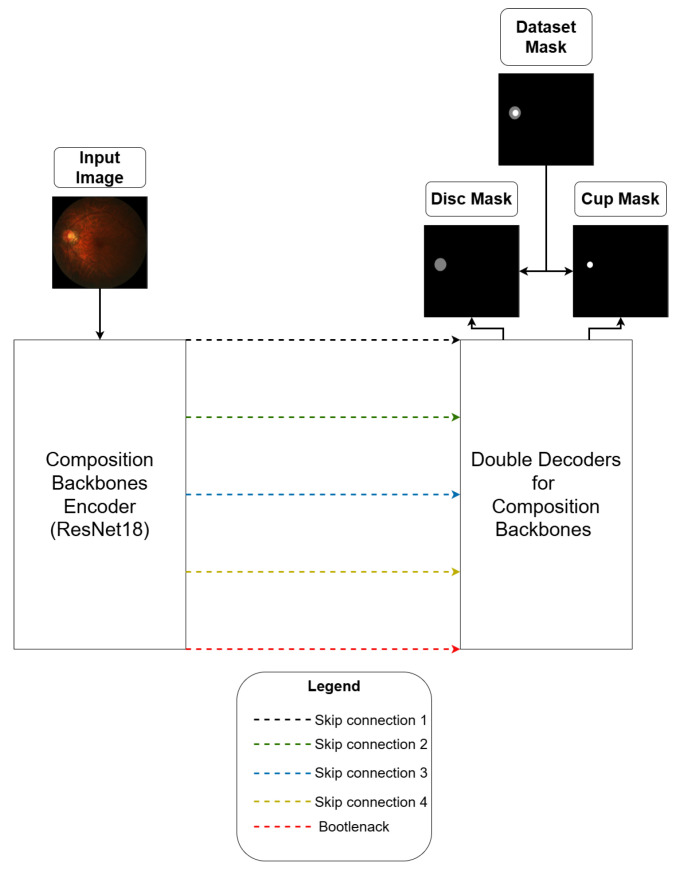
Proposed architecture overview.

The architecture was inspired by [28], and an overview can be seen in Figure 2. Moreover, as a similarity, an architecture based on U-Net was chosen.

**Figure 3:**
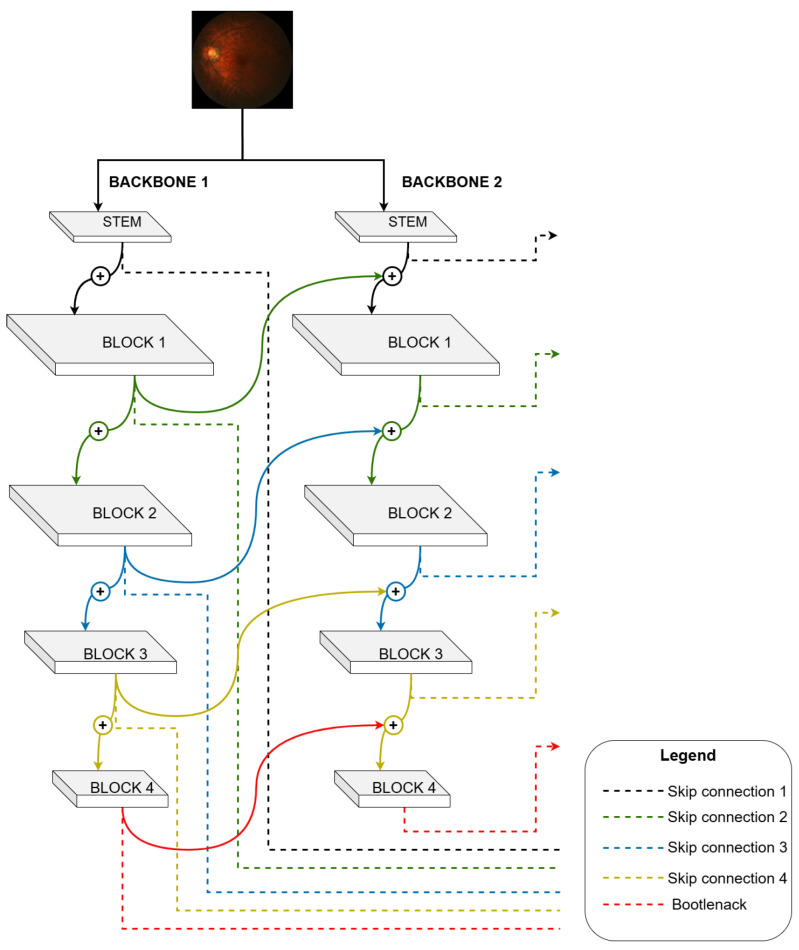
Architecture’s backbones.

The developed architecture uses the Same-Level Composition (SLC) model. It has two backbones, where the stem block is a sequential with 3 × 3 convolution, batch normalization, ReLU activation and 2 × 2 max pooling used for low-level feature extraction (Figure 3).

The main idea of this structure is that the composition of multiple pre-trained backbones improves the quality of the features learned by the network without the need for additional pre-training because, similar to other ensemble methodologies, the backbone composition uses different networks to calculate the prediction maps. However, unlike traditional ensemble methods, in this approach, there is no need for voting or weighting between the components since only the last backbone is responsible for the model’s prediction map [28]. In its operation, a backbone composition resembles a Recurrent Convolutional Neural Network (RCNN), which uses recurrent connections in the convolution layers. However, unlike RCNN, there are no bidirectional connections in the backbone composition, being a complete feedforward architecture [28].

Resnet18 was chosen as the pre-trained backbone because it is a lighter structure than transformers and Efficientnet and has good results in segmentation tasks.

**Figure 4:**
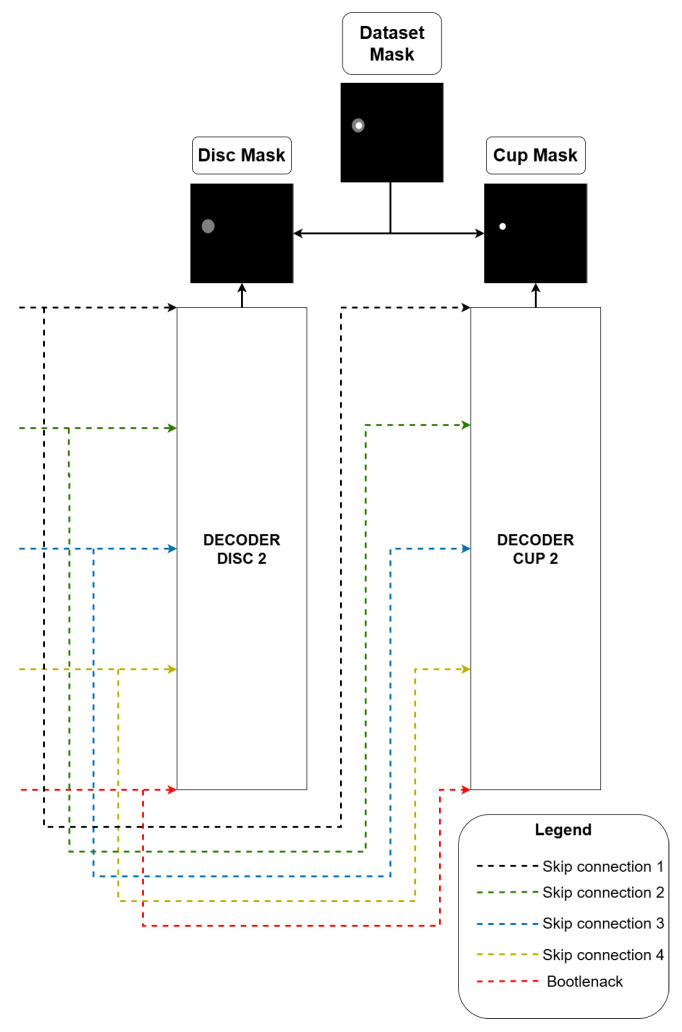
Architecture’s decoders.

The backbone composition model is susceptible to the gradient loss problem, as it increases the number of trainable layers in the model. A solution to mitigate this problem is proposed, using a decoder layer connected to each backbone, as seen in Figure 3, formed by two backbones; two decoder layers are used, where one decoder is used as an auxiliary in training (auxiliary loss), and the other decoder is used as model output. Each decoder comprises two components, as seen in Figure 4.

Consider the weighted sum of the losses of all backbone/decoder pairs, according to Equation (1).

(1) $$L=L_{\mathrm{Lead}}+\sum_{i=1}^{b-1}\left(\lambda_{i}\cdot L_{\mathrm{Assist}}^{i}\right)$$

where L is the model loss, $L_{\mathrm{Lead}}$ is the last backbone loss, $\lambda_{i}$ is the weight of each i-th backbone, and $L_{\mathrm{Assist}}^{i}$ is the loss of the i-th backbone.

This approach was applied with a ratio of 0.5 for each weight of each backbone, starting with backbone 1 (leftmost in the model) to backbone b (rightmost in the model). Thus, the greatest weight is that of the backbone, whose decoder is used as the model output. However, we propose a new way of calculating the model loss, according to Equation (2). Furthermore, for the calculation of the factors, λ values were obtained by Equation (3), and for the calculation of *z* in the *softmax* function of Equation (3), a series obtained by Equation (4) was used.

(2) $$L=\lambda_{b}\cdot L_{\mathrm{Lead}}+\sum_{i=1}^{b-1}\left(\lambda_{i}\cdot L_{\mathrm{Assist}}^{i}\right)$$

where $\lambda_{b}$ is the weight of the last backbone.

(3) $$\lambda_{i}=\frac{e^{z_{i}}}{\sum_{j=1}^{b}e^{z_{j}}},\;for\;i=1,2,3,\ldots ,b$$

(4) $$z_{i}=i\times factor,\;for\;i=1,2,3,\ldots ,b$$

where *factor* is an input parameter and *i* is the backbone index in the model.

The best value for the *factor* was 0.3 in the experiments performed.

### 3.4. Evaluation

As an evaluation metric for the proposed architecture, the Dice and IoU were used, as they are measures widely used in medical image segmentation evaluations. In particular, the Dice and IoU are the overlaps between two binary regions; they are defined by Dice in Equation (5) and IoU in Equation (6), where X is the ground truth and Y is the predicted truth [29].

(5) $$DICE=\frac{2\times (X\cap Y)}{X\cup Y}$$

(6) $$IoU=\frac{X\cap Y}{X\cup Y}$$

where *X* is the ground truth pixel values and *Y* is the predicted pixel values.

## 4. Results

To evaluate this architecture, as seen in Section 3, the dataset BrG was used. The images were used to train and test the architecture with k folds (five folds).

The experiments were conducted on a computer with a Core i5 12400F processor, 16GB of RAM, and a single NVIDIA GeForce RTX 3060 GPU with 12GB of VRAM. Python 3.12.9 was used with PyTorch 2.5.1 framework, Albumentations, OpenCV and Sckit-learn libraries.

Because the architecture has adjustable hyperparameter values (learning rate, batch size, class balance factor, and loss function balance factor) and aims to evaluate the best-fitted model, the optimization of these values was performed using the Optuna framework [30]. In this step, 200 possible combinations were evaluated, and the best set of hyperparameters used in the architecture training and testing process were selected.

The methods for calculating loss were BinaryCrossEntropy loss and Dice loss for each class (optic disc and optic cup). They were chosen because they are widely used in the medical image segmentation domain.

In the training and testing process, the dataset was divided into five parts, with three folds used in training, one in validation and the other in testing, according to the k-fold methodology. This division was executed five times.

Dice and IoU indexes were computed using test data for each fold. As a result, an average dice index of 96.03% was obtained for OD and 85.11% for OC, and an average IoU index of 92.42% was obtained for OD and 75.42% for OC. All Dice and IoU k-folds results are presented in Table 2.

### Discussion

When we analyze Table 2, we observe lower deviation rates between folds, indicating the method’s stability independently of the validation fold. The standard deviation at IoU OD is 0.33%, IoU OC is 0.70%, the standard deviation at Dice OD is 0.19%, and the Dice OC is 0.62%.

**Figure 5:**
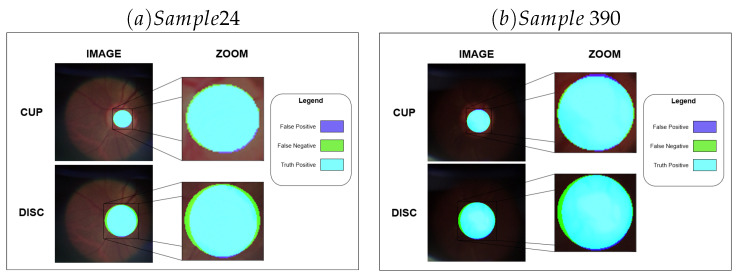
Examples where the method demonstrates a high value of Dice.

**Figure 6:**
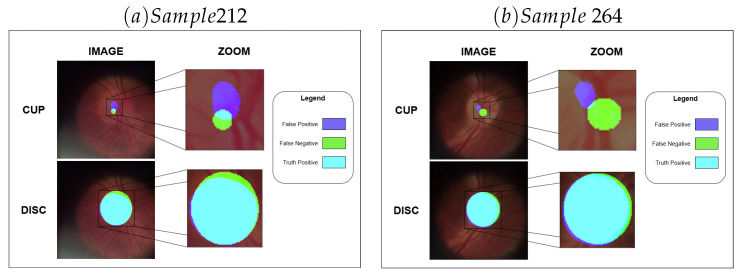
Examples where the method did not capture the pattern.

The results also demonstrate a significant difference between the fidelity of representation of the OD about the OC, which may be caused by the difference in the size of the disc and cup structures, especially in healthy individuals. Moreover, this assumption becomes stronger when observing the individual results of segmentations with high results, as exemplified in Figure 5, and with low results, as exemplified in Figure 6.

Upon closer examination of the positive results, it becomes evident that minor prediction errors occur at the boundaries of the region of interest. This pattern is observable in Figure 5.

When the analysis is performed on the most significant prediction failures, there are several situations, such as the prediction of a region being more minor than it is, as in Figure 6a, or even cases in which the sizes and positions are wrongly predicted, as in Figure 6b.

To better analyze the results, we propose discussing case studies and evaluating the results over GRADCAM and performing other comparisons with baseline networks and the ORIGA dataset.

Experiments were performed with baseline architectures to compare the results obtained from the proposed architecture. We performed this test because BrG is still a recent dataset and has not yet been used in cup-and-disc segmentation tasks. So we wanted to evaluate the quality of the constructed model. The networks used were U-Net [31], Deeplab V3 [32] and Segformer [33], as they are widely used in segmentation tasks and obtain good results in this task. The results are presented in Table 3.

We observe a gain in segmentation quality of the Composite Encoder Double Decoder in comparison with the other baseline architectures, both with the OD, where the average increase is 8.65% in the Dice index and 14% in the IoU index, and with the OC, where the average increase is 0.69% in the Dice index and 0.81% in the IoU index.

The results obtained in U-Net, Deeplab V3, and Segformer present a standard deviation close to those obtained in Composite Encoder Double Decoder, which may indicate the heterogeneity of the BrG dataset. As in the Composite Encoder Double Decoder, the remaining architectures present better results in segmenting the OD concerning the OC, which may indicate difficulty detecting the OC boundaries in the dataset’s images.

The proposed method was also applied to the ORIGA [34] dataset. The database comprises 650 retinographic exams with their respective segmentation masks. The dataset is an annotated subset from retinal images collected on SiMES [35], a study about risk factors for blindness in the Singapore Malay community. All images from ORIGA are 2048 pixels in height and width, between 2301 and 2764 pixels. The ORIGA dataset was also used with k folds (five folds) to obtain a baseline reference.

We want to evaluate the stability of the proposed method with this test, even if the images were acquired from different perspectives and with different methods. The result was an average Dice index of 96.05% for OD and 86.37% for OC, and an average IoU index of 92.05% for OD and 76.86% for OC. All Dice and IoU results k-folds are presented in Table 4.

The same behavior, shown in the BrG dataset, can be observed in the tests performed on the ORIGA dataset, where the standard deviation in IoU OD is 0.43%, the IoU OC is 1.19%, and the standard deviation in Dice OD is 0.23% and Dice OC is 0.85%. At this point, it is essential to emphasize that the two datasets are different, and therefore, it can be assumed that the model can obtain a good representation of the OC and OD structures. The results indicate similar results when performed with the BrG dataset and demonstrate that the proposed network could reach promising results independently of the dataset.

We also apply an explainability mechanism to verify the reliability of each backbone’s contribution to the model’s final result as an auxiliary method for evaluating the proposed model. For this purpose, the SEG-GRAD-CAM presented by [36], an extension of the Grad-CAM proposed by [37], was used.

Grad-CAM is widely used as an explainability method for image classification tasks because it presents, in heat maps, the most significant weights of the activation maps in a given layer of the deep network. This attribute provides the designer of deep models and users of these models with a tool for interpreting the response given by the model [37]. SEG-GRAD-CAM uses the principles of Grad-CAM to assemble heat maps for a given segmentation class C. Thus, by applying SEG-GRAD-CAM N times, where N is the number of classes, and with each application being oriented to one of the N classes, it is possible to interpret which points of the activation maps were used for the model’s response for each class [36].

To evaluate and analyze the backbone outputs in each composite backbone architecture, SEG-GRAD-CAM was applied to the output of Block 4, which corresponds to the backbone output for the input of the architecture’s decoder flow. Thus, the network with two backbones generates four Grad-CAM heatmap images, one pair (OD, OC) for each output of the two backbones of the model.

**Figure 7:**
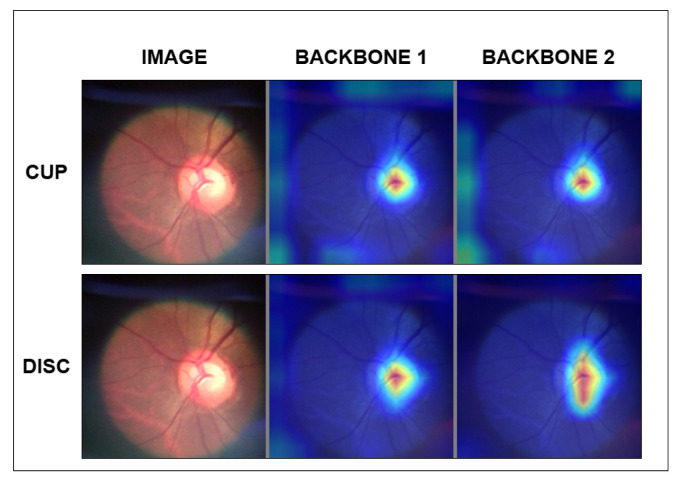
Grad-CAM of sample 24.

When observing the activation maps presented in Grad-CAM of successful case segmentations, as in Figure 7, we can perceive a behavior of persistence and reinforcement of the quality of the segmentations of the structures. Disc activation maps best exemplify this behavior. This may indicate why the network can delimit the OD region with greater precision. However, when we analyze the cups’ activation maps, we observe activation regions at the edges of the image. This behavior may explain why the model does not achieve better results in delimiting the cup regions.

**Figure 8:**
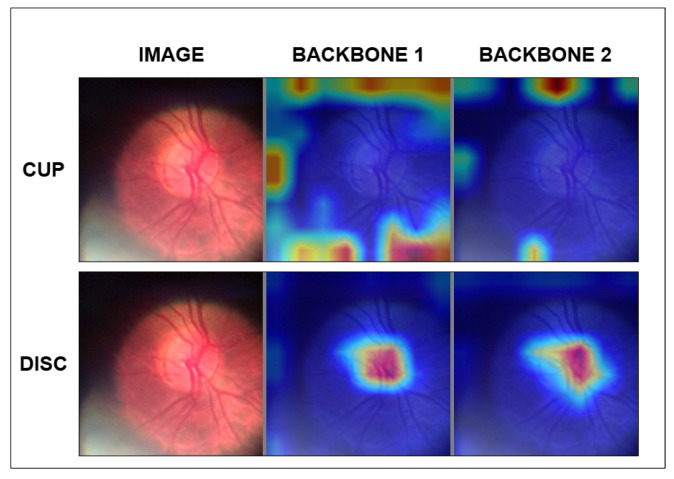
Grad-CAM of sample 212.

Another important factor to be perceived in the Grad-CAM activation maps is acquisition failures, as occurred in Figure 8, where in the lower-left corner of the sample, there is a clear region, possibly resulting from light entering the ophthalmoscope at the time of the examination. This region generated activations in regions that do not belong to the optic nerve region and may contribute to a reduction in the precision of the OC and OD contours.

When observing the activation maps of a case with low Dice rates, we notice that the architecture backbones tend to activate more at the edges, highlighting a behavior already observed in the samples with better results. This can be seen in Figure 8, where no activation maps exist in the disk regions.

## 5. Conclusions

Because it leads to irreversible damage, glaucoma requires early diagnosis to avoid blindness. A low-cost patient screening process can optimize disease detection, enabling early treatment. Applying an automatic detection tool using ophthalmoscopic images obtained by smartphones through a deep learning architecture can be fundamental in this process, especially in underdeveloped countries with a shortage of equipment and professionals. Thus, the Composite Encoder Double Decoder architecture was presented to fill this gap.

The results obtained in experiments with the proposed architecture demonstrate that the backbone composition with a double decoder can improve the quality of segmentation of optic nerve structures in preliminary experiments on the ORIGA dataset, reaching 95.81% for OD and 86.37% for OC in the Dice indexes, of 92.05% for OD and 76.86% for OC in the IoU index. In the BrG dataset, which is a set of images obtained by a smartphone coupled to a panoptic ophthalmoscope, the architecture performs with 95.92% for OD and 85.30% for OC with Dice indexes of 92.22% for OD and 75.68% for OC in IoU index, on the BrG dataset and presenting a real gain in segmentation quality compared with the results of state-of-the-art architectures in the segmentation task. In this way, the proposed model can effectively be used in screening processes, allowing studies into the possibility of creating prevention programs in the primary care environment.

However, other parts of this architecture can be improved. The decoder and the segmentation head were formed only by convolutional layers, and the binary cross-entropy and data were the loss functions applied. This construction allows these other components to be replaced by structures more suitable for this activity. Using a graph-based sub-architecture in the decoder can allow a more profound understanding of the characteristics of the ocular structures in the fundus images. A segmentation head considering the objects’ contours can help refine the generated segmentation’s quality. It is also a loss function that does not predominantly consider the error in the center of the object but rather the contour error. Finally, the evaluation of this model in a screening environment in a primary care program should be conducted to obtain concrete data on its application. 

## Figures and Tables

**Table 1:** Summary of related works.

	Proposition	Cup Dice	Disc Dice	Cup IoU	Disc IoU	Datasets
[12]	FC-DenseNet network	86.59%	96.53%	76.88%	93.34%	ORIGA, DRIONS-DB, Drishti-GS, ONHSD, RIM-ONE
[13]	Resnet 34 Encoder with 2 Steps	88.77%	97.38%	80.42%	94.92%	RIGA, DRISHTI-GS, RIM-ONE
[14]	Multi-Scale Attention UNet	93.4%	96.4%	87.5%	92.8%	REFUGE, ORIGA
[15]	U-Net with Residual and Attention Mechanisms	93.48%	97.48%	87.77%	95.09%	REFUGE, RIM-ONE, Drishti-GS
[16]	EE-TransUNet	90.68%	97.74%	84.10%	95.59%	RIM-ONE, REFUGUE, DRISHTI-GS
[17]	RMHA-Net	87.87%	95.15%	86.75%	85.28%	Drishti-GS, ORIGA, PAPILA, Chaksu, REFUGE
[18]	Attention-based with dense dilated series convolutions	88.7%	95.95%	79.72%	92.22%	REFUGE, PAPILA, ORIGA, Drishti-GS, G1020, CRFO
[19]	Patch-Based Output Space Adversarial Learning	88.26%	96.02%	—	—	DRISHTI-GS, RIM-ONE, REFUGE
[8]	Framework based on Fast R-CNN	90.27%	96.34%	—	—	REFUGE, ORIGA
[20]	Graph Convolutional Network	95.58%	97.76%	91.60%	95.64%	REFUGE, Drishti-GS
[21]	Sector Association and Multi-Coordinate Transformation Fusion	90.32%	96.20%	—	—	REFUGE, Drishti-GS, private dataset from Beijin Tongren Hospital
[22]	HR-Net with Contour Reconstruction	91.78%	97.65%	—	—	ORIGA, DRISHTI-GS
[23]	Ensemble Learning	89.4%	96.1%	80.8%	92.5%	REFUGE, RIM-ONE, Drishti-GS
[24]	Post processing with edge detection	90.2%	96.5%	82.4%	93.3%	Drishti-GS, ORIGA, RIM-ONE, REFUGE
[25]	Unsupervised domain adaptation	95.44%	87.63%	—	—	RIGA+, REFUGE
[26]	Single-source domain generalization	83.07%	93.71%	—	—	RIGA+, REFUGE

**Table 2:** Results from the BrG dataset.

Brazil Glaucoma Dataset				
	Dice		IoU	
Fold	OD	OC	OD	OC
1	95.81%	85.59%	92.05%	75.85%
2	96.09%	85.69%	92.53%	76.18%
3	95.92%	85.30%	92.22%	75.68%
4	96.01%	84.72%	92.38%	74.90%
5	96.31%	84.23%	92.92%	74.49%
**Average**	**96.03%**	**85.11%**	**92.42%**	**75.42%**

**Table 3:** Comparison results on the BrG dataset.

Results with Baseline Architectures				
	Dice		IoU	
Network	OD	OC	OD	OC
U-Net	86.96%	84.61%	77.74%	74.86%
Deeplab V3	87.27%	84.60%	78.22%	74.79%
Segformer	86.98%	84.56%	77.79%	74.87
Composite Encoder Double Decoder	95.92%	85.30%	92.22%	75.68%

**Table 4:** Results obtained in ORIGA dataset.

ORIGA Dataset				
	Dice		IoU	
Fold	OD	OC	OD	OC
1	96.03%	86.56%	92.48%	77.24%
2	95.52%	87.44%	91.51%	78.20%
3	95.98%	86.40%	92.33%	76.86%
4	95.59%	85.07%	91.66%	74.93%
5	95.91%	86.36%	92.26%	77.07%
**Average**	**95.81%**	**86.37%**	**92.05%**	**76.86%**

## Data Availability

The original contributions presented in the study are included in the article. Further inquiries can be directed to the corresponding authors.
